# Optimisation of Operational Conditions during the Production of *Arthrospira platensis* Using Pilot-Scale Raceway Reactors, Protein Extraction, and Assessment of their Techno-Functional Properties

**DOI:** 10.3390/foods11152341

**Published:** 2022-08-05

**Authors:** Silvia Villaró, Ainoa Morillas-España, Gabriel Acién, Tomás Lafarga

**Affiliations:** 1 Department of Chemical Engineering, University of Almeria, 04120 Almeria, Spain; 2 Functional Unit Desalination and Photosynthesis, CIESOL Solar Research Centre, 04120 Almeria, Spain

**Keywords:** microalgae, Spirulina, photobioreactor, surface response methodology, proteins, functional properties, cyanobacteria

## Abstract

The aim of the present study was to identify the optimum combination of dilution rate and depth of the culture to maximise the *Arthrospira platensis* BEA005B (Spirulina) productivity using 80 m^2^ raceway reactors. By varying these two main operational conditions, the areal biomass productivity of the reactors varied by over 55%. The optimum combination, optimised using a surface response methodology, was a depth of 0.10 m and a dilution rate of 0.33 day^−1^, which led to a biomass productivity of 30.2 g·m^−2^·day^−1^ on a dry weight basis when operating the reactors in semi-continuous mode. The composition of the produced biomass was 62.2% proteins, 42.5% carbohydrates, 11.6% ashes, and 8.1% lipids. The isolated proteins contained all the essential amino acids (except for tryptophan, which was not determined); highlighting the content of valine (6.8%), histidine (8.3%), and lysine (7.5%). The functional properties of the proteins were also assessed, demonstrating huge potential for their use in the development of innovative and sustainable foods.

## 1. Introduction

The microalgae *Arthrospira platensis* and *Arthrospira maxima*, commercially known as Spirulina, are the most widely produced microalgal strains. Spirulina has been collected from lakes and consumed by humans for centuries. Because of its long history of use, Spirulina can be consumed in the European Union (EU) with no need to comply with Regulation (EU) 2015/2283 known as the Novel Foods Regulation. This, together with the high protein content of Spirulina, has led to an important increase in the number of products containing Spirulina that are launched to the market [1].

Spirulina is produced using photobioreactors located inland. Microalgal photobioreactors can be divided into two major groups: closed and open systems. Because of their ease of scale up and maintenance and their lower operational and fixed costs, raceway designs are the most common photobioreactor designs [2]. It is estimated that 90% of worldwide microalgal biomass production is done using raceway reactors [3]. These reactor designs have some limitations associated with their relatively low areal productivities, challenging control, and risk of contamination. The latter is especially challenging, and for this reason only a limited number of strains can be produced outdoors during long periods of time. These strains have some common and key characteristics: they are robust and fast-growing, produce and accumulate a valuable compound, and grow in extreme environments. For example, the microalga *Dunaliella salina* accumulate β-carotene and can cope with salinity concentrations higher than 60 g·L^−1^ [4]. These conditions minimise the appearance of bacteria, fungi, rotifers, and other algae that are algal predators or compete with *D. salina* for nutrients. Spirulina is another example of a microalga that can be produced outdoor because of its tolerance to high alkaline conditions. The optimum pH value for Spirulina growth is in the range 9–10, where bicarbonate prevails in the bicarbonate/carbonate equilibrium [5]. This microalga can also cope with relatively high temperatures, with the optimum growth temperature somewhere between 30 and 35 °C [6], and produce and accumulate large quantities of proteins with all the essential amino acids [7]. Spirulina also produce phycobiliproteins, which are bright blue (phycocyanin and allophycocyanin) or fuchsia (phycoerythrin) water-soluble proteins that can be used in the nutraceutical, cosmetic, or pharmaceutical industries [8].

Most of the Spirulina consumed in the EU is imported from Asia, where microalgae are produced in large open ponds. However, the productivity of these systems has not been reported. Moreover, despite being the most widely produced microalga, most of the data available in the scientific literature was obtained using laboratory-scale photobioreactors, most of them located indoors and completely controlled, which are not representative of commercial environments. Up to the best of the authors knowledge, only one study carried out in Brazil from 2005 to 2006 evaluated the efficiency of pilot-scale raceway reactors (37 m^2^, 10 m^3^) operated in semi-continuous mode and reported an average biomass productivity of 21.6 g·m^−2^·day^−1^ [9]. In that study, the strain used was Spirulina sp. LEB-18, the depth of the culture was 0.5 m, and 25% of the cultures volume was harvested every 72 h. More pilot- and large-scale works are needed to demonstrate the potential of producing Spirulina in the EU and promote private investments. Moreover, it is important to identify how operational conditions affect biomass productivity and therefore, maximise the efficiency of current reactor designs. The most common and easily adjustable operational conditions in raceway reactors are the dilution rate and the depth of the culture. The depth of the culture is directly related with light availability. The higher the cultures’ depth, the lower the light availability because of the self-shading effect of microalgae. Moreover, the dilution rate is also related with light availability but also with nutrient availability. The higher the dilution rate, the higher the light availability because more biomass is daily removed and therefore the biomass concentration is lower [10].

The aim of the present study was to use a response surface methodology (RSM) to identify the optimum combination of dilution rate and depth of the culture for pilot-scale raceway reactors operated following industrial practices. The reactors (80 m^2^) were located inside a greenhouse in Almería (Spain) and the produced biomass was characterised in terms of macromolecular composition. Moreover, the produced biomass was used to generate a protein isolate whose amino acid profile and techno-functional properties were determined.

## 2. Materials and Methods

### 2.1. Microalgal Strain and Culture Medium

The microalga studied was *A. platensis* BEA 005B, purchased from the Spanish Bank of Algae (Las Palmas, Spain). The inocula were produced initially using 80 L pH-controlled bubble columns, and then using three identical 1.2 m^3^ raceway photobioreactors described elsewhere [10]. In both cases, a culture at a concentration of approximately 1.3 g·L^−1^ was used as inoculum. The bubble columns and the raceway reactors were operated in batch mode until the concentration was constant for at least three consecutive days (8–9 days of operation). The medium used for biomass production was developed using commercial fertilisers: 0.90 g·L^−1^ NaNO_3_, 0.14 g·L^−1^ KH_2_PO_4_, 0.18 g·L^−1^ MgSO_4_·7H_2_O, 15 mg·L^−1^ CaCl_2_·2H_2_O, 16.8 g·L^−1^ NaHCO_3_, and 30 mg·L^−1^ of Karentol^®^ (Konegard, Barcelona, Spain), a commercial mixture of agricultural micronutrients.

### 2.2. Biomass Production

**Figure 1:**
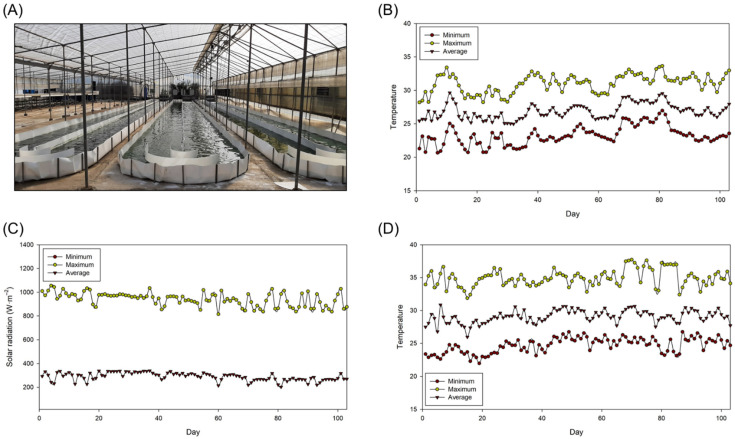
(**A**) Photobioreactors used, (**B**) temperature inside the greenhouse, (**C**) solar radiation that reached the culture, and (**D**) temperature of the culture during biomass production. Maximum and minimum values refer to the maximum and minimum temperature or solar radiation values measured every day. The average temperature values refer the average temperature during the 24 h and the average solar radiation values refer to the average irradiance during the illuminated period.

The biomass was produced using the 80 m^2^ raceway reactors shown in Figure 1. These reactors were located inside a greenhouse at the SABANA Demonstration Plant in Almería (Spain) and were described elsewhere [11]. In the present study, the depth of the culture varied between 10 and 20 cm; therefore, the total volume of the culture varied from 9 to 17 m^3^. The reactors were inoculated with 10% of their working volume (0.9–1.7 m^3^) using the inocula described in the previous section, and then filled up to the final working volume using the culture medium described above. The reactors were operated in batch mode until the biomass concentration of the culture was steady for at least three consecutive days. Then, the reactors were operated in semi-continuous mode using a dilution rate that varied from 0.15 to 0.45 day^−1^.

The fresh culture medium introduced into the system was the same used for preparing the inocula with the exception of NaHCO_3_, which was added on demand. The reactors were operated 24 h per day; the pH was kept constant at 9.8 ± 0.1 by on-demand injection of CO_2_. Freshwater was added to the reactor every day to compensate the evaporation losses. The environmental conditions were monitored as well as the reactors that were online monitored and controlled using a SCADA system and the data is available at the database http://sabana.ual.es/ (accessed on 4 August 2022).

The biomass concentration, nutrient concentration, and chlorophyll fluorescence were daily determined during the semi-continuous operation. Briefly, the biomass concentration was determined gravimetrically after filtration and oven-drying in an oven at 80 °C during 24 h. The biomass was washed with tap water twice to remove the excess of salts before filtration. The biomass productivity was calculated on a dry weight basis as the product of the dilution rate and the biomass concentration of the culture. The Fv/Fm value of the culture was determined using an AquaPen AP 100 fluorimeter (Photon System Instruments, Drásov, Czech Republic). The determination was carried out after 10 min of dark adaptation. The concentrations of N-NO_3_^−^ and P-PO_4_^3−^ were determined spectrophotometrically using a Genesys 10S spectrophotometer (Thermo Fisher Scientific, Barcelona, Spain) as described elsewhere [12]. After three weeks of semi-continuous operation, the culture was harvested using an industrial SSD 6-06-007 centrifuge (GEA Westfalia Separator, Oelde, Germany) and the biomass was washed with tap water twice to remove the excess of salts. Approximately 1 kg of microalgal paste was vacuum-sealed and stored at −20 °C until further analysis. Moreover, another 1 kg of microalgal paste was dried using a Mobile Minor^®^ PSR spray dried (GEA Westfalia Separator, Oelde, Germany) and the dried powder was stored in vacuum-sealed bags at −20 °C until further use.

### 2.3. Experimental Design

The optimisation of the main operational conditions was carried out using a response surface methodology with Design Expert v11 (Stat-Ease Inc., Minneapolis, MN, USA). The effect of the two independent variables, which were the depth of the culture and the dilution rate, on the biomass productivity of the raceway reactors, expressed on a dry weight basis, was investigated using a central composite face-centred design. The software generated 11 experimental runs that are listed in Table 1.

The central point was repeated tree times to assess the error within the model. The dilution rate varied between 0.15 and 0.45 day^−1^. This value refers to the amount of culture that is daily harvested and replaced with fresh culture medium; for example, a dilution rate of 0.15 day^−1^ implies that 15% of the total volume of the culture was harvested every day and replaced with fresh culture medium. In addition, the depth of the culture, which has a direct impact on light availability, varied from 10 to 20 cm. The response was the biomass productivity of the system, determined as the product of the biomass concentration and the dilution rate used. The experimental data were fitted to a polynomial response surface, which was the best fitted model, using the equation:

(1) $$Y=\beta_{0}+\sum_{i=1}^{n}\beta_{i}X_{i}+\sum_{i=1}^{n}\beta_{ii}X_{i}^{2}+\sum_{i=1}^{n}\sum_{j=i+1}^{n}\beta_{ij}X_{i}X_{j},$$

where Y is the dependent variable, $\beta_{0}$ is the centre point of the system, $\beta_{i}$, $\beta_{ii}$, and $\beta_{ij}$ are the coefficients of the linear, quadratic, and interactive effect, and $X_{i}$, $X_{i}^{2}$, and $X_{i}X_{j}$ are the linear, quadratic, and interactive effect of the independent variables. The non-significant terms were deleted from the polynomial model after analysis of variance (*p* < 0.05) and a new analysis of variance was carried out to obtain the final coefficients of the equation. The developed model was validated and optimised in triplicate using the same reactors.

### 2.4. Assessment of the Macromolecular Composition

The crude protein was determined using a LECO FP628 protein analyser (LECO Corp., St. Joseph, MI, USA). The total protein content was estimated from the total nitrogen content using a nitrogen-to-protein conversion factor of 5.95 [13]. The total lipid content was calculated gravimetrically; the lipids were extracted following the Folch method using chloroform:methanol (2:1 *v*/*v*) [14]. The ash content was determined by calcination of the biomass at 600 °C for 12 h [15]. The carbohydrate content was determined as the residual weight after substracting the amounts of water, protein, fat, and ash.

### 2.5. Protein Isolation

The proteins were isolated from the wet microalgal paste following an ultrasound-assisted solubilisation/precipitation methodology described elsewhere [16]. The only difference with that study is that in the present work, the precipitated proteins were dialysed against distilled water using 3.5 kDa molecular weight-cut off SnakeSkin^TM^ Dialysis Tubing (Thermo Scientific, Waltham, MA, USA). The isoelectric point at which the majority of the proteins were recovered was 3.9 and the pH was adjusted using either 1 M NaOH or 1 M HCl (0.1 M for fine adjustment). The centrifugation was carried out using a Sigma 3-18 KS centrifuge (Sigma Laborzentrifugen GmbH, Osterode am Harz, Germany). The isolated proteins were freeze-dried and stored vacuum-sealed at −20 °C until further analysis.

### 2.6. Amino Acid Profile

The amino acid profile was assessed as described elsewhere [17]. Briefly, 10 mg of dried biomass were hydrolysed using 10 mL of 6 M HCl at 110 °C for 24 h. Then, the hydrolysate was filtered using 0.45 µm filters and evaporated to dryness under nitrogen at 40 °C. The dried residue was suspended in 2 mL of distilled water and the amino acids were quantified using a Perkin Elmer Series 200 HPLC (PerkinElmer, Waltham, MA, USA) coupled to a Perkin Elmer Altus A-10 fluorescence detector (PerkinElmer, Waltham, MA, USA). Each natural replicate was determined in duplicate.

### 2.7. Assessment of Techno-Functional Properties

The techo-functional properties of the isolated proteins namely their solubility, their foaming (FC) and emulsifying capacities (EC), their water- (WHC) and oil-holding capacities (OHC) were assessed using previously described methods [18]. The solubility of the proteins at different pH values was measured by means of the Lowry method as described elsewhere [13] using BSA as the standard. The homogenisation was carried out using a T-25 digital ULTRA-TURRAX (IKA, Staugen, Germany) and the centrifugation steps were performed using a Sigma 3-18 KS centrifuge (Sigma Laborzentrifugen GmbH, Osterode am Harz, Germany).

### 2.8. Statistical Analysis

Unless mentioned otherwise, all the analytical determinations were conducted in triplicate per natural replicate. The statistical differences were analysed using ANOVA with JMP 13 (SAS Institute Inc., Cary, NC, USA) and Tukey HSC tests were used to identify where sample differences occurred. The criterion for statistical significance was *p* < 0.05.

## 3. Results and Discussion

### 3.1. Biomass Production

Environmental conditions have a significant effect on the productivity of photosynthetic organisms. However, in the present study, the average values of solar radiation and temperature inside the culture were comparable during the different experimental runs. Indeed, the average solar radiation on the surface of the culture and the average temperature inside the greenhouse during all the experimental period were 290.5 ± 33.6 µmol·m^−2^·s^−1^ and 26.9 ± 1.1 °C, respectively. The environmental conditions during the experimental period led to average, maximum, and minimum temperature values of the culture of 29.1 ± 0.9, 34.9 ± 1.4, and 24.7 ± 1.1 °C, respectively. These conditions were adequate for production the selected strain as the Fv/Fm values determined during the semi-continuous production of the biomass ranged from 0.5 to 0.6, which demonstrate that the cultures were not subjected to stress conditions such as an excess of light, lack of nutrients, or the presence of toxins [19].

**Figure 2:**
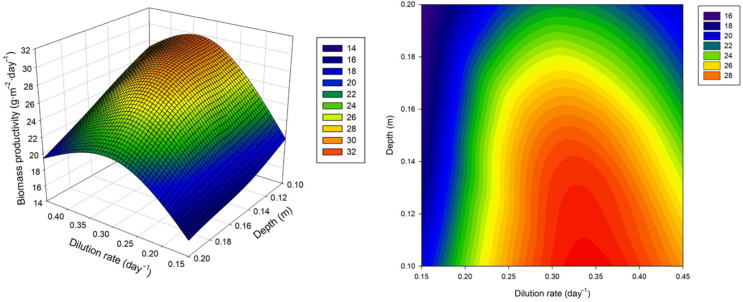
Effect of dilution rate and culture depth on biomass productivity.

The biomass productivity achieved operating the reactors at variable dilution rates and culture depths is listed in Table 1. The results were expressed on a dry weight basis. Overall, the biomass productivity varied between 15.9 ± 0.8 and 29.3 ± 0.1 g·m^−2^·day^−1^, which means that varying the main operational conditions allowed increasing the productivity of the system by 45%. If up-scaled to a theoretical 1 ha facility, and assuming that the environmental conditions are within the same range during 90 days, the production capacity of the system during summer would increase from 14.3 to 26.4 tn. Figure 2 displays the 3D and 2D contour model graphs generated from the results of the experimental runs, showing the combinations of operational conditions that led to higher areal biomass production values.

The biomass productivity was modelled using a central composite face-centred design. The data, the biomass productivity, could be fitted to a polynomial quadratic equation:

(2) $$Biomass\;productivity\;\left(g\cdot m^{-2}\cdot day^{-1}\right)=4.037-0.623\cdot h+197.711\cdot D-298.222\cdot D^{2}$$

where h and D are the depth of the culture, expressed in cm, and the dilution rate, expressed in day^−1^, respectively. This equation represent an empirical relationship between the biomass productivity of the utilised raceway reactors and the independent variables, which were the operational conditions dilution rate and depth of the culture. It is important to remark that those variables and interactions between variables that were not significant were not considered. The statistical analysis revealed that the quadratic model for biomass productivity was adequate (*p* < 0.0005) and with a good determination coefficient (R^2^ = 0.9518). The model F-value of 46.06 (*p* < 0.0001) implies that the proposed model is significant. The Lack of Fit F-value of 8.87 indicated that the Lack of Fit is not significant relative to the pure error, which means that the model fits within the design space. In addition, the predicted R^2^ of 0.8678 was in reasonable agreement with the adjusted R^2^ of 0.9311. The Adequate Precision value was 20.31. This parameter measures the signal to noise ratio; the results indicated an adequate signal and suggested that the model can be utilised to navigate the design space.

Both the dilution rate and the depth of the culture had a significant effect on biomass productivity (*p* < 0.0001). As highlighted before, both parameters have a striking effect on light availability, which is one of the most important factors affecting the production of photosynthetic organisms. The linear term of the cultures’ depth was negative, which means that an increase in the depth of the culture leads to a decrease in biomass productivity. This is supported by the fact that thin-layer cascade photobioreactors that are operated at much lower culture depths (0.5–5.0 cm) are more productive than conventional raceways [20]. Other works also demonstrated that lower culture depths in raceway reactors increase biomass productivity [21]. The combined dilution rate and depth of the culture that led to a higher biomass productivity was determined. Overall, higher biomass productivities were observed when operating the reactors at a depth of the culture around 10 cm and a dilution rate close to 0.3 day^−1^. The optimum dilution rate is strain-dependent. However, previous studies using the microalga *Scenedesmus almeriensis* also suggested that the optimal dilution rate in the region is around 0.3 day^−1^ [12]. To obtain the highest biomass productivity, the optimal dilution rate and depth of the culture were predicted to be 0.33 day^−1^ and 10 cm, respectively. For these conditions, the predicted biomass productivity was 30.56 g·m^−2^·day^−1^ and the Desirability value was 0.709. The nearest the Desirability value is to the unit, the more adequate the system is [22]. Finally, the predicted as optimum operational conditions (10 cm and 0.33 day^−1^) were validated against experimental results obtaining a biomass productivity value of 30.2 ± 0.4 g·m^−2^·day^−1^, which was in good agreement with the predicted value.

### 3.2. Optimisation of the Culture Medium

**Figure 3:**
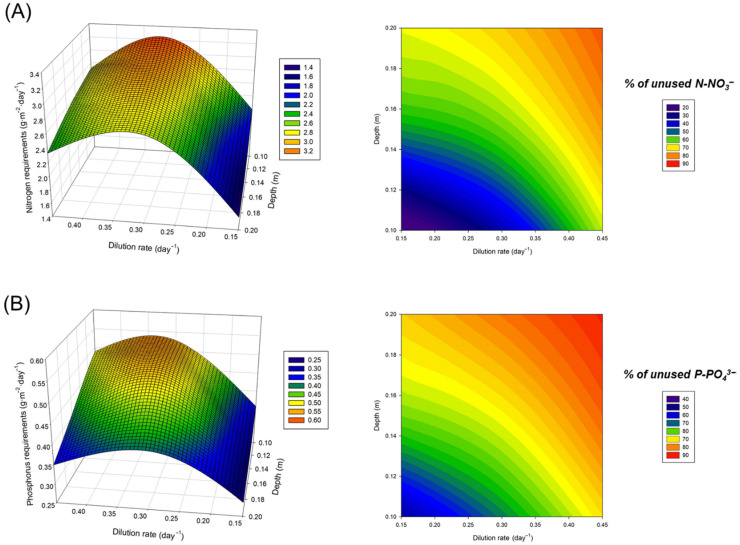
Effect of dilution rate and culture depth on the requirements and utilization of (**A**) N-NO^3−^ and (**B**) P-PO_4_^3−^.

The present study evaluated the effect of operational conditions on the productivity of Spirulina. Not only operational conditions but also environmental conditions and the composition of the culture medium can affect the productivity of the system as well as the composition of the produced biomass. As highlighted above, the environmental conditions were similar during the different experimental runs and it is unlikely that the observed effects were caused by weather changes. Moreover, the same culture medium was used for all the different experimental runs. The culture medium contained inorganic nitrogen (N-NO_3_^−^) and phosphorous (P-PO_4_^3−^) needed for microalgal growth. These two elements are the main nutrients of concern in eutrophication, which causes algal blooms and the degradation of water bodies [23]. Minimising nutrient pollution from agriculture (and human activities) is a key priority. Therefore, the culture medium used for microalgae production should aim at minimising the nitrogen and phosphorus concentration in the inlet and outlet effluents as much as possible. The model described above was developed using both nutrients in quantities that ensured that the culture would not be nutrient-limited. This also promoted the production and accumulation of proteins, which was the ultimate goal of producing Spirulina. It has been demonstrated that nitrogen-limitation in microalgal cultures produces the degradation of proteins and the production and accumulation of carbohydrates and lipids [24]. However, after measuring the content of nitrogen and phosphorus in the produced biomass and the N-NO_3_^−^ and P-PO_4_^3−^ concentration of the outlet effluents, a mass balance revealed that a significant proportion of the total nitrogen (35–85%) and phosphorus (35–90%) that entered the system was left unused. Both the dilution rate and the depth of the culture had a striking effect on the amount of nutrients that were left unused (Figure 3). The reason for this is that the N-NO_3_^−^ and P-PO_4_^3−^ available in the media was assimilated by microalgae to produce biomass and both operational conditions had a significant influence on biomass productivity as discussed above. For this reason, based on the biomass productivity that was achieved when operating the reactors at a dilution rate of 0.33 day^−1^ and at a depth of 10 cm (30.2 g·m^−2^·day^−1^), a different medium was prepared assuming that the nitrogen and phosphorus content of the biomass would be 10 and 2%, respectively. This medium contained 0.54 and 0.08 g·L^−1^ of NaNO_3_ and KH_2_PO_4_, respectively, representing a 40 and 43% decrease from the concentration in the original recipe. These values were a 10% higher than the theoretical values required to ensure that the culture will not be limited. The biomass productivity achieved when operating the reactors using this novel medium was 29.7 ± 0.7 g·m^−2^·day^−1^, which was comparable to that obtained when using higher nutrient concentrations (30.2 g·m^−2^·day^−1^).

The results demonstrated that reducing the nitrogen and phosphorus content of the culture medium by approximately 40% the biomass productivity of the system was not affected. Moreover, the macromolecular composition of the produced biomass was also not affected, with comparable concentrations of proteins, lipids, ashes and carbohydrates (Table 2).

The reduction in the nutrient use would not only reduce the environmental impact of microalgae production but also save money for microalgae producers, as nutrients represent approximately 24% of the costs associated with raw materials [25]—although this value depends largely on the nutrient source used. Water also contributes to the sustainability and economic viability of microalgal processes. It is important to highlight that industrial production of the biomass should always recirculate the water back into the system to minimise the water requirements of microalgae production, which are approximately 1000 L of process water per 1 kg of biomass [26]. For this, the use of ultrafiltration membranes is a novel approach that should be encouraged and further studied as only a limited number of works assessed their potential implementation in microalgal processes [27].

### 3.3. Protein Extraction and Assessment of Techno-Functional Properties

Spirulina is well known for its high protein content, which is generally around 60% on a dry weight basis [28]. In the present study, the produced biomass had a high protein content (62.2%), which is in line with previous works [29]. Spirulina proteins are especially interesting because of their content in essential amino acids in line with the FAO and WHO composition of an “ideal” protein [7]. In the present study, the protein isolate obtained following an ultrasound-assisted solubilisation/precipitation strategy (and purified using dialysis) had a protein content of 81.3 ± 0.4 g·100 g^−1^. The amino acid content of the isolated proteins is listed in Table 3.

The essential amino acids are those that cannot be synthesised by the human body and must come from food. The produced biomass had all the essential amino acids (except for tryptophan, which was not determined). When compared to the composition of other conventional protein sources such as egg white, soybean or pea, summarised elsewhere [30], the content of valine (6.8%), histidine (8.3%), and lysine (7.5%) in the isolated proteins was especially high.

**Figure 4:**
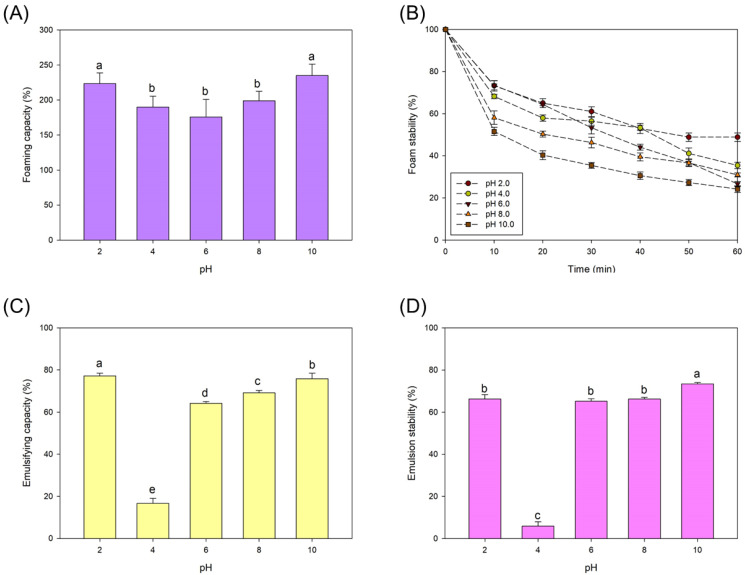
(**A**) Foaming capacity, (**B**) foam stability, (**C**) emulsifying capacity, and (**D**) emulsion stability. Different letters indicate significant differences (*p* < 0.05).

The WHC and OHC of the protein isolate was 2.11 ± 0.13 and 2.96 ± 0.23 g·g^−1^. Both the WHC and OHC of proteins affect the functionality and sensorial properties of foods: high WHC values contribute to maintain moisture in, for example, baked products and high OHC values can improve flavour and palatability of, for example, meat analogues [18]. The foaming and emulsifying capacities of the proteins are shown in Figure 4. The FC was high at all the studied pH values, especially at pH 2.0 and 10.0 (*p* < 0.05). The higher FC at low and high pH values can be attributed to a higher protein solubility. Moreover, the FS was higher at pH 2.0 and 4.0, which can be attributed to the formation of thick and elastic interfacial films at pH values close to the pI of the protein, which in the present study was 3.9. The EC and the ES of the isolated proteins are shown in Figure 4.

Both parameters were higher at a pH value above or below the pH of the proteins, where the net charge of the proteins is zero and there are fewer interactions between the protein and water. The low EC and FC values at a pH value of 4.0 were caused by a low solubility of the proteins, which were isolated by precipitation at a pH 3.9. The results reported in the present study are comparable to those presented in previous works that demonstrated that proteins derived from Spirulina show huge potential for being used in the manufacture of innovative foods. For example, an *A. platensis* protein isolate obtained from commercial Spirulina demonstrated high water- and oil-holding capacities [31]. In that same study, the maximum FC and EC values were 255 and 65%, respectively. In a different work, a protein concentrate (76% protein) from *A. platensis* LEB 52 that contained all the essential amino acids showed foaming properties similar to those of egg white. The maximum FC and EC values were 226 and 84%, respectively [32]. The present study produced the microalga *A. platensis* BEA005B at a scale of 80 m^2^ and following industrial manufacturing practices. Moreover, the extraction methods used to produce the protein isolates were not the same and both factors could explain the differences between these two reports and the present study. However, results demonstrate the potential of using Spirulina-derived proteins as novel techno-functional ingredients. Other works suggested Spirulina-derived proteins as potential sources of bioactive hydrolysates and peptides [2].

## 4. Conclusions

The present study demonstrated the technical viability of producing Spirulina in the EU using pre-commercial scale raceway reactors. The results demonstrated that operating the reactors using dilution rates or culture depths different from the optimum could reduce the productivity of the system by 50%. It is important to optimise the operation of microalgal photobioreactors, and the depth of the culture and the dilution rate can be easily optimised and adjusted. The concentration of nitrogen and phosphorus in the culture medium can also be optimised by reducing their content to a level that allows for maximum growth and ensures that the culture is not nutrient-limited, but minimising the amount of nutrients that are left unused. The Spirulina produced in the present study had a high protein content, comparable to that of commercial edible Spirulina. These proteins contained all the essential amino acids (except for tryptophan, which was not determined) and a functionality that was comparable to that of other proteins being commercially used in the food industry. It is likely that because of the sustainable nature and the high functionality of Spirulina-derived proteins, the number of products containing Spirulina that are launched into the market will continue to grow.

## Figures and Tables

**Table 1:** Central composite response surface design for biomass productivity.

	Coded Variables		Actual Variables		Response
Run ^a^	Depth	Dilution Rate	Depth (cm)	Dilution Rate (day^−1^)	Productivity (g·m^−2^·day^−1^)
1	−1	0	10	0.30	29.3
2	0	0	15	0.30	28.1
3	1	0	20	0.30	22.2
4	1	−1	20	0.15	15.9
5	1	1	20	0.45	19.4
6	0	0	15	0.30	27.6
7	−1	−1	10	0.15	19.8
8	−1	1	10	0.45	27.1
9	0	0	15	0.30	28.6
10	0	−1	15	0.15	17.2
11	0	1	15	0.45	23.3

^a^ The experimental run number does not correspond to the order of production, which was conducted randomly.

**Table 2:** Biomass composition.

	Medium I ^a^	Medium II ^a^
Lipids (g·100 g^−1^)	8.10 ± 0.85	8.00 ± 0.53
Ashes (g·100 g^−1^)	11.63 ± 0.87	11.81 ± 0.88
Carbohydrates (g·100 g^−1^)	42.46 ± 1.17	41.13 ± 2.95
Proteins (g·100 g^−1^)	62.2 ± 1.77	60.9 ± 1.76

^a^ Medium I refers to the culture medium formulated using 0.90 g·L^−1^ NaNO_3_ and 0.14 g·L^−1^ KH_2_PO_4_ and Medium II refers to the culture medium formulated using 0.54 and 0.08 g·L^−1^ of NaNO_3_ and KH_2_PO_4_, respectively.

**Table 3:** Amino acid profile of the Spirulina-derived protein isolate. Results are expressed as g of amino acid per 100 g of protein.

Name	Concentration (g·100 g^−1^)
Asp–D	7.54 ± 0.37
Thr–T *	3.53 ± 0.28
Ser–S	3.35 ± 0.14
Glu–Q	10.45 ± 0.93
Gly–G	3.09 ± 0.29
Ala–A	4.57 ± 0.31
Cys–C	0.88 ± 0.33
Val–V	6.81 ± 0.29
Met–M *	0.85 ± 1.05
Ile–I *	5.30 ± 0.65
Leu–L *	5.43 ± 0.21
Tyr–Y	4.73 ± 0.40
Phe–F *	5.99 ± 0.27
His–H *	8.34 ± 0.07
Lys–K *	7.53 ± 0.44
Arg–R	5.65 ± 0.40
Pro–P	3.22 ± 0.41

* Essential amino acid.

## Data Availability

The data that supports these findings are available at the SABANA Demonstration Plant database at http://sabana.ual.es/ (accessed on 4 August 2022).

## References

[1] Lafarga T., Fernández-Sevilla J.M., González-López C., Acién-Fernández F.G. (2020). Spirulina for the food and functional food industries. Food Res. Int..

[2] Lafarga T., Sánchez-Zurano A., Villaró S., Morillas-España A., Acién G. (2021). Industrial production of spirulina as a protein source for bioactive peptide generation. Trends Food Sci. Technol..

[3] Barceló-Villalobos M., Guzmán Sánchez J.L., Martín Cara I., Sánchez Molina J.A., Acién Fernández F.G. (2018). Analysis of mass transfer capacity in raceway reactors. Algal Res..

[4] Zhu Q.L., Bao J., Liu J., Zheng J.L. (2020). High salinity acclimatization alleviated cadmium toxicity in *Dunaliella salina*: Transcriptomic and physiological evidence. Aquat. Toxicol..

[5] Ragaza J.A., Hossain M.S., Meiler K.A., Velasquez S.F., Kumar V. (2020). A review on Spirulina: Alternative media for cultivation and nutritive value as an aquafeed. Rev. Aquac..

[6] Colla L.M., Oliveira Reinehr C., Reichert C., Costa J.A.V. (2007). Production of biomass and nutraceutical compounds by *Spirulina platensis* under different temperature and nitrogen regimes. Bioresour. Technol..

[7] Morist A., Montesinos J.L., Cusidó J.A., Gòdia F. (2001). Recovery and treatment of *Spirulina platensis* cells cultured in a continuous photobioreactor to be used as food. Process Biochem..

[8] Pagels F., Guedes A.C., Amaro H.M., Kijjoa A., Vasconcelos V. (2019). Phycobiliproteins from cyanobacteria: Chemistry and biotechnological applications. Biotechnol. Adv..

[9] Morais M.G., Radmann E.M., Andrade M.R., Teixeira G.G., Brusch L.R.F., Costa J.A.V. (2009). Pilot scale semicontinuous production of Spirulina biomass in southern Brazil. Aquaculture.

[10] Sánchez-Zurano A., Morillas-España A., Gómez-Serrano C., Ciardi M., Acién G., Lafarga T. (2021). Annual assessment of the wastewater treatment capacity of the microalga Scenedesmus almeriensis and optimisation of operational conditions. Sci. Rep..

[11] Morillas-España A., Lafarga T., Sánchez-Zurano A., Acién-Fernández F.G., Rodríguez-Miranda E., Gómez-Serrano C., González-López C.V. (2021). Year-long evaluation of microalgae production in wastewater using pilot-scale raceway photobioreactors: Assessment of biomass productivity and nutrient recovery capacity. Algal Res..

[12] Morillas-España A., Lafarga T., Gómez-Serrano C., Acién-Fernández F.G., González-López C.V. (2020). Year-long production of Scenedesmus almeriensis in pilot-scale raceway and thin-layer cascade photobioreactors. Algal Res..

[13] López C.V.G., del Carmen Cerón García M., Fernández F.G.A., Bustos C.S., Chisti Y., Sevilla J.M.F. (2010). Protein measurements of microalgal and cyanobacterial biomass. Bioresour. Technol..

[14] Perera E., Sánchez-Ruiz D., Sáez M.I., Galafat A., Barany A., Fernández-Castro M., Vizcaíno A.J., Fuentes J., Martínez T.F., Mancera J.M. (2020). Low dietary inclusion of nutraceuticals from microalgae improves feed efficiency and modifies intermediary metabolisms in gilthead sea bream (*Sparus aurata*). Sci. Rep..

[15] García-Márquez J., Rico R.M., Sánchez-Saavedra M.D.P., Gómez-Pinchetti J.L., Acién F.G., Figueroa F.L., Alarcón F.J., Moriñigo M.Á., Abdala-Díaz R.T. (2020). A short pulse of dietary algae boosts immune response and modulates fatty acid composition in juvenile *Oreochromis niloticus*. Aquac. Res..

[16] Sánchez-Zurano A., Morillas-España A., González-López C.V., Lafarga T. (2020). Optimisation of protein recovery from *Arthrospira platensis* by ultrasound-associated isoelectric solubilisation/precipitation. Processes.

[17] Bongiorno T., Foglio L., Proietti L., Vasconi M., Lopez A., Pizzera A., Carminati D., Tava A., Vizcaíno A.J., Alarcón F.J. (2020). Microalgae from biorefinery as potential protein source for Siberian sturgeon (*A. baerii*) aquafeed. Sustainability.

[18] Garcia-Vaquero M., Lopez-Alonso M., Hayes M. (2017). Assessment of the functional properties of protein extracted from the brown seaweed *Himanthalia elongata* (Linnaeus) S. F. Gray. Food Res. Int..

[19] Ciardi M., Gómez-Serrano C., Morales-Amaral M.D.M., Acién G., Lafarga T., Fernández-Sevilla J.M. (2022). Optimisation of *Scenedesmus almeriensis* production using pig slurry as the sole nutrient source. Algal Res..

[20] Masojídek J., Sergejevová M., Malapascua J.R., Kopecký J., Prokop A., Bajpai R., Zappi M. (2015). Thin-layer systems for mass cultivation of microalgae: Flat panels and sloping cascades. Algal Biorefineries: Products and Refinery Design.

[21] Sánchez-Zurano A., Lafarga T., Morales-Amaral M.D.M., Gómez-Serrano C., Fernández-Sevilla J.M., Acién-Fernández F.G., Molina-Grima E. (2021). Wastewater treatment using *Scenedesmus almeriensis*: Effect of operational conditions on the composition of the microalgae-bacteria consortia. J. Appl. Phycol..

[22] Odriozola-Serrano I., Soliva-Fortuny R., Martín-Belloso O. (2009). Impact of high-intensity pulsed electric fields variables on vitamin C, anthocyanins and antioxidant capacity of strawberry juice. LWT Food Sci. Technol..

[23] Anderson D.M., Glibert P.M., Burkholder J.M. (2002). Harmful algal blooms and eutrophication: Nutrient sources, composition, and consequences. Estuaries.

[24] Li X., Li W., Zhai J., Wei H. (2018). Effect of nitrogen limitation on biochemical composition and photosynthetic performance for fed-batch mixotrophic cultivation of microalga *Spirulina platensis*. Bioresour. Technol..

[25] Acién F.G., Fernández J.M., Magán J.J., Molina E. (2012). Production cost of a real microalgae production plant and strategies to reduce it. Biotechnol. Adv..

[26] Murphy C.F., Allen D.T. (2011). Energy-water nexus for mass cultivation of algae. Environ. Sci. Technol..

[27] Morillas-España A., Sánchez-Zurano A., Lafarga T., del Mar Morales-Amaral M., Gómez-Serrano C., Acién-Fernández F.G., González-López C.V. (2021). Improvement of wastewater treatment capacity using the microalga *Scenedesmus* sp. and membrane bioreactors. Algal Res..

[28] da Rosa G.M., Moraes L., Cardias B.B., de Souza M.D.R.A.Z., Costa J.A.V. (2015). Chemical absorption and CO_2_ biofixation via the cultivation of Spirulina in semicontinuous mode with nutrient recycle. Bioresour. Technol..

[29] Aouir A., Amiali M., Bitam A., Benchabane A., Raghavan V.G. (2017). Comparison of the biochemical composition of different Arthrospira platensis strains from Algeria, Chad and the USA. J. Food Meas. Charact..

[30] Gorissen S.H.M., Crombag J.J.R., Senden J.M.G., Waterval W.A.H., Bierau J., Verdijk L.B., van Loon L.J.C. (2018). Protein content and amino acid composition of commercially available plant-based protein isolates. Amino Acids.

[31] Benelhadj S., Gharsallaoui A., Degraeve P., Attia H., Ghorbel D. (2016). Effect of pH on the functional properties of Arthrospira (Spirulina) platensis protein isolate. Food Chem..

[32] Lupatini Menegotto A.L., de Souza L.E.S., Colla L.M., Costa J.A.V., Sehn E., Bittencourt P.R.S., de Moraes Flores É.L., Canan C., Colla E. (2019). Investigation of techno-functional and physicochemical properties of Spirulina platensis protein concentrate for food enrichment. LWT.

